# Encouraging Spontaneous Synchronisation with D-Jogger, an Adaptive Music Player That Aligns Movement and Music

**DOI:** 10.1371/journal.pone.0114234

**Published:** 2014-12-09

**Authors:** Bart Moens, Chris Muller, Leon van Noorden, Marek Franěk, Bert Celie, Jan Boone, Jan Bourgois, Marc Leman

**Affiliations:** 1 Institute for Psychoacoustics and Electronic Music, Department of Musicology, Ghent University, Ghent, Belgium; 2 Department of Management, University of Hradec Králové, Hradec Králové, Czech Republic; 3 Department of Movement and Sports Sciences, Ghent University, Ghent, Belgium; UNLV, United States of America

## Abstract

In this study we explore how music can entrain human walkers to synchronise to the musical beat without being instructed to do so. For this, we use an interactive music player, called D-Jogger, that senses the user's walking tempo and phase. D-Jogger aligns the music by manipulating the timing difference between beats and footfalls. Experiments are reported that led to the development and optimisation of four alignment strategies. The first strategy matched the music's tempo continuously to the runner's pace. The second strategy matched the music's tempo at the beginning of a song to the runner's pace, keeping the tempo constant for the remainder of the song. The third alignment starts a song in perfect phase synchrony and continues to adjust the tempo to match the runner's pace. The fourth and last strategy additionally adjusts the phase of the music so each beat matches a footfall. The first two strategies resulted in a minor increase of steps in phase synchrony with the main beat when compared to a random playlist, the last two strategies resulted in a strong increase in synchronised steps. These results may be explained in terms of phase-error correction mechanisms and motor prediction schemes. Finding the phase-lock is difficult due to fluctuations in the interaction, whereas strategies that automatically align the phase between movement and music solve the problem of finding the phase-locking. Moreover, the data show that once the phase-lock is found, alignment can be easily maintained, suggesting that less entrainment effort is needed to *keep* the phase-lock, than to *find* the phase-lock. The different alignment strategies of D-Jogger can be applied in different domains such as sports, physical rehabilitation and assistive technologies for movement performance.

## Introduction

Many people like to perform sports activities such as walking and running while listening to music. Recent studies indeed provide evidence that music can empower people, showing a strong effect on perceived exertion and performance boosts, especially when movements are synchronised with the musical beat, and when music has a motivating character [1]–[4]. However, many aspects of this phenomenon of musical empowerment have not been fully understood and further study is needed, especially in the light of interactive technologies and their applications.

In this paper we focus on the interactive technology, called D-Jogger, which allows for a mutual synchronisation of a person and a music player. With this interactive technology we wanted to explore how people entrain (i.e. adapt their synchronisation behaviour) to music players by controlling parameters such as the tempo and phase of the music played. We are interested in finding the optimal control parameters for inducing a *spontaneous entrainment* that would lead to a stable synchronisation between person and music player.

Up until quite recently, beat-synchronised walking or running was only possible when the person was explicitly instructed to adapt to the fixed beat played by the music player. It was shown in [5] that without instruction to synchonise, spontaneous gait synchronisation did not occur during treadmill walking. However, several studies show that instructed beat-synchronisation may encourage running and walking [1], [2], [6]–[12]. In some of these studies, the tempo of each song was fixed and a playlist was chosen in advance to match the person's tempo as close as possible to encourage the synchronisation.

With advances in technology, it is now possible to use a music player that automatically synchronises the tempo of the song to the gait frequency, or even synchronise each beat of the music to a footfall. D-Jogger [13] is a music player which, from a playlist of songs of differing tempi, is able to increase or decrease the speed or pulse of a song to match a listeners' walking or running pace. The tempi of the music will be in step with the progression of the person's movements, making incremental adjustments as the person advances. However, if the subject would suddenly change tempo; for example, from running to walking, such that the difference in tempo exceeds the range of adaptation of the initial tempo, then D-Jogger would select another song nearer to the person's new tempo. The tempo of the song can then adjust to match the person's tempo. Even more interesting, perhaps, is that the D-Jogger can also change the phase of a song so that it begins together with the footfall. The phase can also be adapted after a pause in motion. Several other devices, methods, and settings have been proposed, such as walking in synchrony with a robot [14], walking in synchrony with music in recreational settings [15]–[17], running with music in sport performance settings [6], [9], [10] and incorporating using music in rehabilitation training programs as rhythmical auditory stimuli (RAS) [18]–[21]. However, D-Jogger is rather unique in its real-time manipulation of phase. This makes the system very adaptive and interactive.

However, a major question deals with the type of adaptation and interaction that is made possible. What strategies induce spontaneous entrainment and stable synchronisation behaviour between a person and an adaptive music player? In addition, can these strategies be optimised?

Despite the fact that an expansive body of literature exists on sensorimotor synchronisation, there is a paucity of knowledge as regards the precise mechanisms that control the mutual adaptation of two interacting rhythms [22], [23]. Repp and Su [24] draw a useful distinction between different paradigms in adaptive (often interpersonal rather than person-machine) entrainment, chiefly the synchronisation-continuation paradigm, and the intentional and non-intentional entrainment. For example, in tapping studies that use the synchronisation-continuation paradigm, effects are observed whereby people mutually adjust their tapping, forming a unit of two-followers, rather than one or of them taking the lead, e.g. [25]. In intentional entrainment, the general observation is that the sensorimotor synchronisation of each person (and by extension of person and machine) relies on mutual temporal error-correction processes that involve continuous phase-correction adjustments. However, it is likely that human adjustment is also based on temporal assimilation, that is, adjustments involving higher-order periodicities implied in the signal [26]. This is especially relevant for music, which is characterised by accents and distributions of energy which promote the emergence of higher-order structures (i.e. metric levels, given beats as stimulus), (see also [4] for acoustical features, and [27] for a theoretical model of attending). In unintentional or spontaneous entrainment, studies often focus on sensorimotor synchronisation activities linked with walking or running. For example, unintentional tempo and phase-locking occurs in pairs of people walking side-by-side on different treadmills [28], or for interlimb coordination when only visual coupling is available [29]. However, between a person and a machine, a stable system in both phase and frequency of tapping was more easily formed when the virtual partner's adaptation rate was slow [30], [31]. Myake [14] also showed that two walking systems (one human and the other a robot) can adapt mutually, and that stable synchronisation can be achieved automatically on the basis of metronome cues. Modelling approaches [32], [33] show that spontaneous entrainment emerges from dynamical laws that operate via mediators on interactions, whereby entrainment is facilitated if certain conditions are fulfilled [34].

Based on the literature, we believe that there may exist an optimal strategy for inducing spontaneous entrainment and maintaining stable synchronisation between a walking/running person and adaptive music player. Our main hypothesis is that a fine-grained control of period and phase of the musical beat may have a great impact on the interaction and, thus, on the entrainment and alignment of the person-machine synchronisation. In the description below, we use the following terminology: *entrainment* - the dynamics that trigger the interaction (between a person and D-Jogger) to a stable synchronisation. *Alignment* - the sustained match between movement and music. An *alignment strategy* is an algorithm that adapts the period and/or phase of the music in response to the period and/or phase of a person's walking or running movement.

The purpose of this paper is to present an overview of the four alignment strategies that have been developed and evaluated in experiments with users, in order to optimise interaction with the D-Jogger. The four experiments vary in terms of the number of participants (10-119), environment (public exhibition versus laboratory, treadmill versus open air), instructions (experiment with treadmill system versus perception of environment during walking), and even mode of movement (walking vs. running vs. both). The experiments were run in sequence and after each experiment, there were indications that we could improve the alignment strategy for further enhancing spontaneous synchronisation. Finally, we settled upon four alignment strategies that differ in the fine details of the manipulation of the timing of the music player. As will be illustrated, interesting observations can be made about the strategies that control entrainment and alignment. Each strategy may be of interest, depending of the application one has in mind. Accordingly, the present paper systematises these explorations *post hoc*, based on the experimental data available, and draws some conclusions about the observations. In the next section ‘Methods’, we describe the basic components of the D-Jogger system and the methods selected for analysis. Then we present the results of four experiments and the associated four alignment strategies. This is followed by an overview of the results and a summary of the main findings. In the ‘Discussion’ chapter, we link these findings with theoretical perspectives and offer some observations on the limitations of our approach.

## Methods

### A smart music player

**Figure 1:**
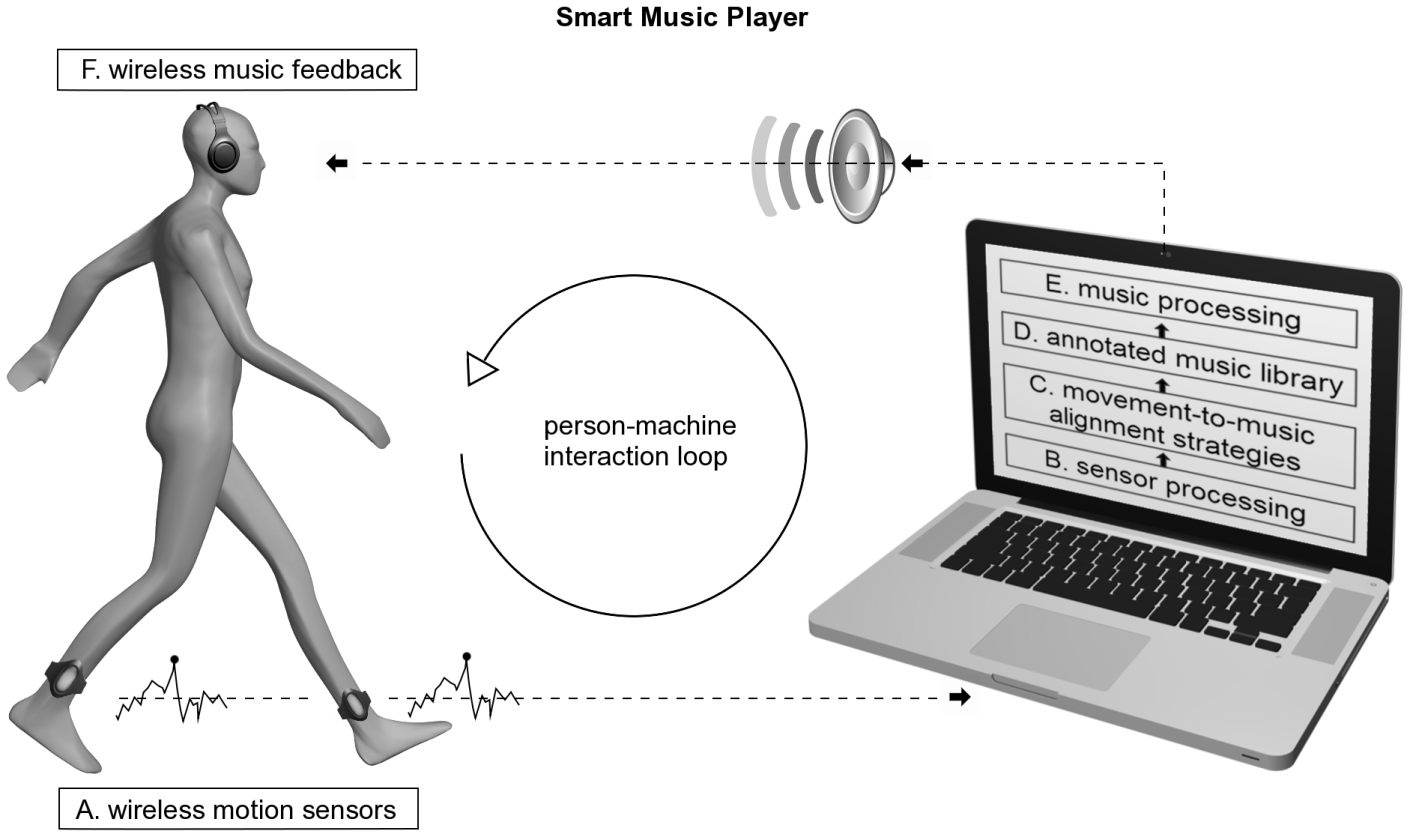
Smart music player: person-machine interaction loop and the main components involved.

D-Jogger is a music player system that adapts the period and phase of a musical playback in response to human interaction. First, the music player identifies the period and phase of a walking or running person, using the footfall as a measurement moment. Based on the selected alignment strategy, music is provided and adapted if needed, using the musical beat as a measurement. Fig. 1 shows the person-machine interaction and the required components. The music player is developed on a portable PC for use in laboratory settings. A feature-limited mobile implementation designed for the Android platform purpose was built for use in outdoor experiments. In the following section we describe the components of the music player.

#### (a) Movement sensor

The system can use a wide range of sensors, capable of measuring the users' movements. The sensors can be attached to various parts of the body: the ankle, the waist (attached to the belt or worn in the pocket), the upper arm, or even slipped inside a shoe sole. The sensors stream data wirelessly to the computer for further analysis. Supported sensors include 3D accelerometers, gyroscopes and pressure sensors.

#### (b) Sensor processing

This component determines the gait frequency and phase using the sensor data. It is based on step detection, using empirically verified algorithms [35]–[37], followed by the extraction of the gait frequency, expressed in steps per minute (SPM), and the gait phase, as determined by the footfall.

#### (c) Alignment strategy

**Figure 2:**
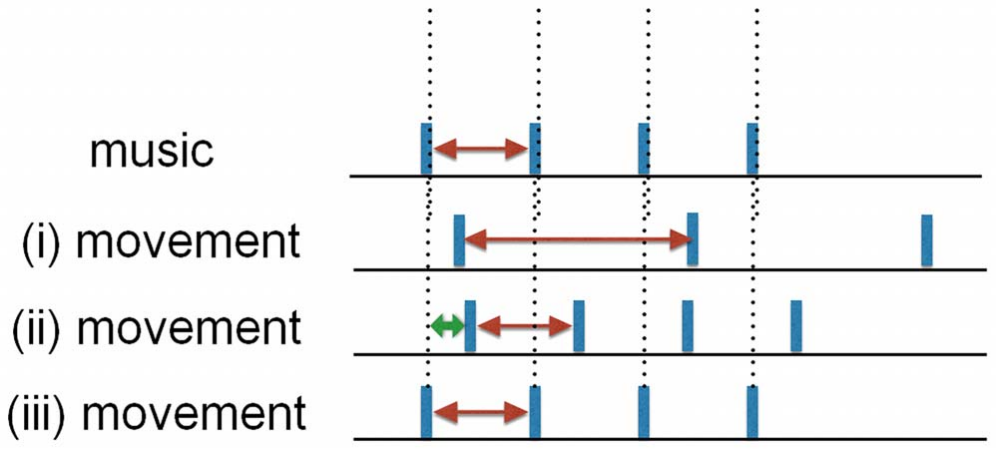
Alignments between music and movement. Given the musical beat shown on top, the movement has (i) no period nor in-phase alignment, (ii) period alignment but no in-phase alignment, (iii) period and in-phase alignment.

This component defines the machine's response to the sensor input, according to the defined alignment strategies. These strategies are based on different elements that are, in a specific way, combined with each other. The basic concepts are period and phase measured at footfall and musical beat. Several alignment situations are shown in Fig. 2. Ultimately, the goal is to achieve a period and in-phase alignment. To that aim, the period of the music can be manipulated to match the person's gait period. The period of the music can be set at the start of a song, using the observed gait period of a person. The phase of the music can be neglected, or manipulated into a certain position with the gait phase. Finally, the start of a song can be delayed so that it begins automatically in phase synchrony with the user. However, once started, the relative phase can be fixed. Based on these elements, period and phase manipulations can be used in conjunction with each other. It is the development of these experiments that has led to these four alignment strategies. More details about the specifics of the strategies can be found in the experiment descriptions.

#### (d) Annotated music library

The system draws from a music database containing a selection of songs and its metadata that consist of the average beats per minute (BPM) and the timings of the beat (phase information). Musical pieces can be added dynamically to this database. They are then processed using an adaption of the Beatroot Algorithm (Mirex 2006 Beat detection winner) [38] in order to create the required metadata, notably tempo and phase information. The system supports manual verification and correction of the metadata.

#### (e) Music processing

This component alters the timing of the music without disturbing the pitch. We use a phase vocoder, which is a real-time music time stretcher (a combination of frequency and time-domain methods) based on the technology Elastique Pitch of ZPlane [39]. It alters the tempo of a song without changing the pitch of the music, making small tempi changes barely audible to the users. We empirically determined that tempo changes of up to +10% and −10% were acceptable in terms of sound quality, resulting in a 20% tempo adjustment range. The tempo adjustment is controlled by the alignment strategies.

#### (f) Music feedback

This component deals with the play back of the manipulated audio to the user.

### Calculating the relative phase of footfall and musical beat

**Figure 3:**
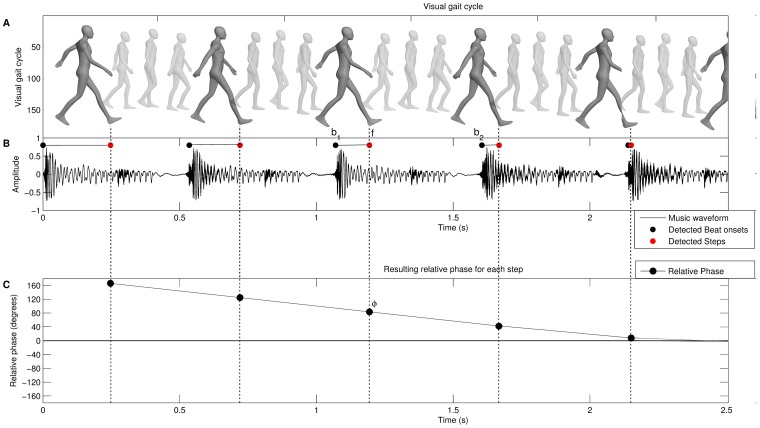
Relative phase of walking on music. Figure A shows the visual gait cycle for left and right steps, figure B shows the waveform of the music with annotated beats. Finally, figure C shows the resulting relative phase which expresses the timing difference between footfalls (A) and musical beat (B).

The relative phase 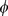 denotes the timing difference between footfall and musical beat in relation to the tempo or period. This difference can be expressed in degrees (from 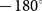 to 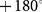). 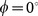 means that the footfall occurred simultaneously with the beat. This is called in-phase or in-sync. 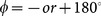 means that the footfall occurred exactly between two consecutive beats; in anti-phase or offbeat. Fig. 3 shows part of a gait cycle that starts near anti-phase and moves towards the in-phase. Left and right footsteps are regarded as equal in terms of relative phase: the phase is calculated for each individual step and is, thus, not based on one complete walking cycle (one left step and one right step). 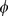 is calculated in realtime using the timing information of two beats and one footfall. Taking 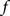 as the moment in time of the footfall for which we want to calculate 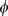; 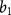 as the time of the onset of the preceding musical beat and 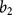 as the time of the succeeding musical beat, then the relative phase is calculated as follows for each footfall: 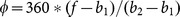. This results in relative phases between 0° and 360°. For visualisation 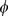 is represented on the −180° to +180° scale (i.e. Fig. 3), so that a negative phase angle indicates that the footfall preceded the musical beat (the footfall is followed by a beat) and vice versa: a positive phase angle indicates the footfall succeeded the musical beat (the footfall occurred right after the beat, see Fig. 3 for an example). When the phase equals 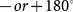, the footfall occurred exactly between the beats.

### Footfalls, system accuracy and delays

It is very difficult to define the precise time of a footfall. This time can be defined as the moment the heel hits the ground (impact, e.g. [36]), the moment pressure is put on the ground (ground reaction force, e.g. [40], [41]) or even the moment of toe touchdown. In our experiments, this moment depends on the type of sensor used, and the location point of where the sensor is attached to the foot. For example, an accelerometer attached at the ankle can easily detect the footfalls using high acceleration peaks to indicate the impact time. A pressure sensor can detect the ground reaction force, making a sharp increase in the signal as an indication of the footfall. In general, the detected footfall timings of these various measurement methods do not register differences up to 25 milliseconds, and we will assume that the detected footfall, regardless of the measurement method, equals the perceived footfall time, as these differences are negligible.

The accuracy of step detection greatly depends on the sample rate of the used sensors. Generally, our sensors use at least a sample rate of 100 Hz (10 milliseconds per sample). However, the actual footfall could occur between two samples resulting in a mean error of 5 milliseconds and 10 milliseconds maximum. Similarly, the used beat tracking algorithm of Beatroot [42] also has an accuracy of 10 milliseconds (for music with a clear audible 4/4 beat), while the actual beat could occur in-between samples. This also results in a mean detection error of 5 milliseconds and 10 milliseconds maximum. Both errors are cumulative, so that at the very least, the total detection error is on average 10 milliseconds. This equals a relative phase error of 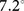 at a frequency of 120 steps per minute, and 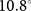 at a frequency of 180 steps per minute. We deemed the accuracy and maximum error sufficient for our real time music system and the subsequent analysis.

In our final remarks, we discuss the delay in the system. Real time gait frequency detection has an inherent delay because at least two steps are needed to determine the tempo. However, the human gait is, in itself, a variable process [43], where gait timings and frequency vary on a step-to-step basis but does revolve around a central frequency. The distribution of gait timings in a healthy walk has a 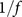-like structure [44]–[46]. Using such unfiltered data results results in significant changes of music tempo per step and could be noticeable to the user. To counteract this behaviour, the gait frequency is determined over multiple steps. This averages out of a part of the variability of the human gait and results in a more stable SPM value. However, footfall timings and phase calculations are not averaged. Both detection methods function independently. When the time between two detected footfalls fails to match the averaged frequency or its harmonics within a small margin, the system assumes an erroneous gait frequency. This prevents false positives in the gait detection. Such false positives are often detected when the user makes a drastic change in the gait pattern, stops, hits an obstacle, touches the sensor, and so forth. Such erroneous phase information is therefore ignored by the algorithms that implement the alignment strategies.

### Statistical methods

Given that phase angles represent directional or circular data, we cannot use standard tests to compare the resulting phase distributions. Consider the following example: the arithmetic mean angle of 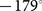 and 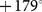 is 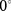, in the framework of our data, the circular mean is 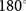 (either seen as 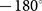 or 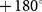). Therefore synchronisation data were analysed with circular statistics [47] using the Circular statistics Toolbox for Matlab [48].

Each alignment strategy results in a distribution of relative phase angles. Such a distribution can be described using the following parameters: the mean angle 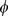 and its confidence interval representing the mean direction, the resultant vector length 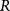 and the circular variance 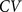 (see further), angular deviation 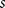 (dispersion around the mean), circular skewness 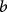 (asymmetry) and the circular kurtosis 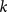 (peak load).

For each distribution, the mean resultant vector 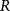 can be calculated [47], [48]. The vector 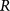 has a phase angle 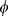 and a length 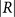. The angle 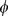 corresponds to the mean relative phase or music synchronisation accuracy and can be interpreted if the distribution of relative phase angles is neither random nor uniform; this is tested with the Rayleigh or Omnibus test. The length 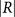 is a measure for synchronicity, which is the mean phase coherence of an angular distribution. 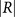 values can range from 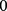 to 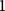, 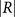 reaches the value 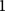 if, and only if, the condition of strict phase locking is adhered to, i.e. all steps were taken in perfect synchrony, whereas 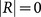 for a uniform distribution of phases, i.e. random phase angles. The circular variance 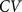 represents the amount of variation in the relative phase angles and is the inverse of the resultant vector length. 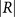 is calculated by 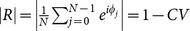, where 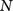 is the number of samples and 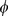 is the relative phase angle for step 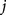.

An angular histogram is used to visually assess the distribution: is it unimodal (single cluster) or multimodal (two or more clusters)? To assess whether participants performed above chance, i.e. whether the alignment algorithm has an influence on the participants behaviour, the Rayleigh test [49] can be used (for unimodal distributions) while the Omnibus or Hodges-Ajne test [50] can be used for multimodal distributions. The null hypotheses for these tests is circular uniformity (random distribution). The alternative hypothesis is a unimodal or multimodal distribution of circular data points centred on a given phase angle. The Rayleigh test additionally tests the significance of the directional mean value, which is not applicable to multimodal distributions (tested with the Omnibus test).

Finally, we analysed the synchronisation stability of individual participants by calculating the resultant vector length and performing a Rayleigh test per participant. When the Rayliegh test reports a significant result 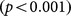, the participant synchronised. We then report the percentage of synchronised participants per experiment.

## The Evaluation of Different Movement-Music Alignment Strategies

An alignment strategy controls the interaction between a person and the machine. In what follows, four alignment strategies will be described and evaluated with an experiment. We describe the experiments and alignments in order of execution: starting from a basic alignment in 2009, to more advanced alignments in 2012. Each experiment builds upon the knowledge and experience learnt from the previous experiments conducted to improve the alignment strategies. Our goal is to maximise uninstructed synchronisation using such an alignment.

**Figure 4:**
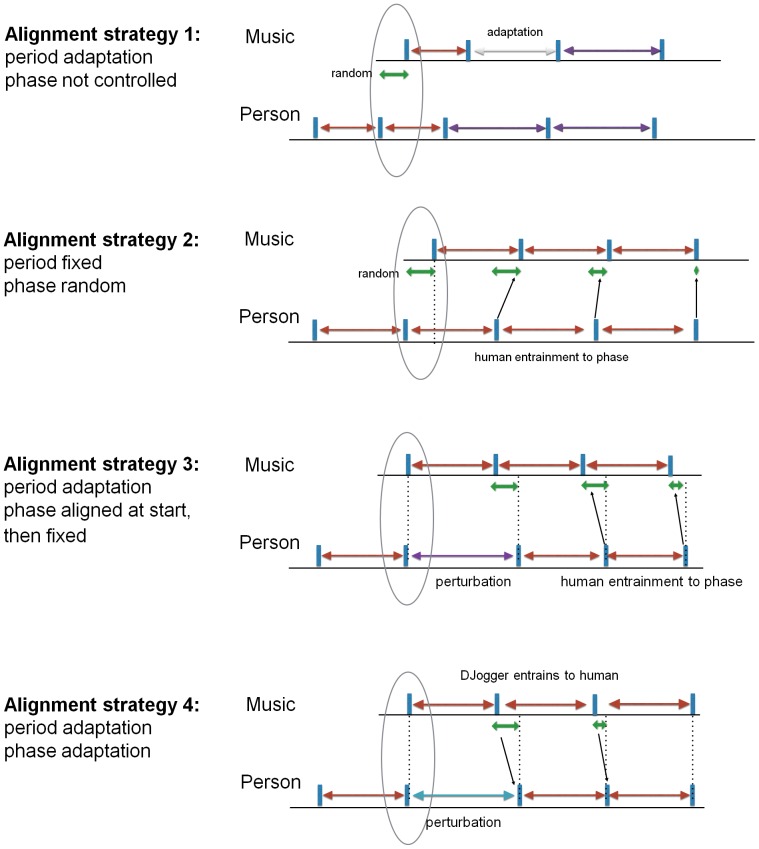
Four different alignment strategies. 1. The song starts in tempo but not in-phase. During the song, the music period is adapted to the walking period. 2. The song starts in tempo but not in-phase. During the song, the music period is kept fixed. The human entrains to the phase of the song. 3. The song starts in tempo and in phase. During the song the phase is kept fixed, while the music period is adapted to the walking period. The human entrains to the phase of the song. 4. The song starts in tempo and in phase. During the song, the period and phase are adapted. The music player follows human changes in period and phase.

Fig. 4 presents an overview of the four different alignment strategies, using beat and footfall as measurement points for timing, music and person. Beat and footfall is represented by the short vertical lines on a horizontal line that represents time. The double arrows on top of a horizontal line represent period, while those below the horizontal line represent relative phase. The oval shape draws attention to the moment where the music begins. In the first two alignment strategies the relative phase is not controlled (and thus random), while in the last two alignment strategies the relative phase is in-phase. The different alignment strategies also suggest which kind of entrainment we may expect. For example, in alignment strategy 2, it is assumed that the person will converge to zero degrees relative phase, similar to that seen in alignment strategy 3. However, in alignment strategy 4, it is the music player that converges its phase with the person. Further details of the alignment strategies are discussed below.

### Alignment strategy 1: period-adaptive phase-random

#### Algorithm description

Our first alignment strategy is rather naive in its approach when it comes to encouraging entrainment. We simply match the tempo of the music (tempo  =  number of musical periods per minute, or Beats Per Minute, or BPM) with the observed tempo of the gait (tempo  =  number of gait periods per minute, or Steps Per Minute, or SPM), as shown in Fig. 4. The relative phase is neglected, which means that the music starts at whatever time interval taken between two steps, and the phase of the music is not adapted during the song. The observed gait tempo (and therefore the music temp) is updated each step and is based on the average inter-step interval of the last 5 steps. Steps are detected using an input sensor and step detection algorithm as described in the previous section. The strategy allows the participant to adjust the relative phase by taking smaller or bigger steps. We denote 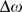 as the value for the difference between the tempo of the musical beat and the tempo of the actual (non-averaged) gait.

The pseudo-code in table 1 describes the alignment strategy and its parameters. The period-adaptive strategy seeks to minimise 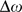. When the participant changes gait tempo, it takes five seconds for the system to realign itself. During this time, 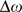 will be non-zero. When the participant holds a steady pace, then 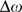 will converge to zero, meaning both frequencies match. When the adjustment needed to minimise 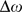 is either smaller than 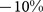 or greater than 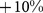, a new song is chosen to prevent an audible deformation of the song. A new song then begins at a random time between footsteps, resulting in a random relative phase at the beginning of a song.

**Table 1:** Algorithm 1.

Pseudocode for alignment strategy 1 (Period-adaptive phase-random)
1: **for all** steps **do**
2: 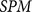 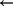 average gait frequency of last 5 steps
3: MusicTempoModifier ( 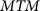 ) 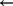 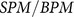
4: **if** 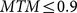 **or** 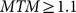 **then** (Tempo outside 10% stretch)
5: **if** for more as 5 seconds **then** (Avoid too many song changes)
6: choose new song ( 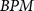 as close to 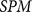 as possible)
7: **end if**
8: **end if**
9: **end for**

#### Hypothesis

We assumed that if the strategy has an influence on the participants' behaviour, then the resulting distribution of the relative phase will be different from a uniform (random) distribution. In addition, there will be a statistically significant mean relative phase angle close to 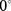, indicating that more then random steps are taken close to the main music beat.

#### Experiment and Participants

Two public demonstrations of the system took place in Belgium and participants volunteered to take part in the experiment with the demonstration system. They gave their consent for their walking behaviour to be recorded. In total, 150 participants were recruited (age  = 21.9 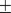 12.9 STD, 82 male, 68 female) of whom 119 participants were included in the analysis. 31 datasets were neglected because the participant spent less then a minute on the treadmill. After a short explanation of the treadmill's operation and safety mechanism, participants could choose to either walk or run on the treadmill. Once the participant was comfortable with the treadmill and its operation, the headphones were given, and the data capture and auditory feedback was set in motion.

#### Ethics statement

The participants signed a consent declaration which stated they had freely volunteered to take part in the experiment and were informed in advance about the task, the procedure and the technology to be used. They were given the opportunity to ask questions and agreed that anonymous recordings of their actions would be made for scientific purposes, only. In keeping with the general standards set by our university and faculty, security was guaranteed (the indoor task posed no risks), and their privacy was respected. According to Belgian law, experiments aimed at research and conducted to further the development of biological or medical knowledge (cf. 7 May 2004 Law concerning experiments on the human body (Ch.II, Art.2, Par.11), is exempt from any requirement to obtain ethical approval. This is because this study only involves behavioural knowledge.

#### Setup

The experimental setup consisted of a basic treadmill, a computer running the smart music player with the above described TA-PR strategy, a Sennheiser HD62TV headphone and a Wii-motion plus a sensor used for step detection. Only one sensor was used per participant, and this was attached comfortably to the right leg above the ankle using elastic Velcro straps. Due to the location of the sensor, footfall impacts were easily detected in real time using peak detection algorithms on the accelerometer signal. The resulting gait frequency was then doubled before transmitting it to the music alignment algorithm. All data was logged for analysis after the experiment.

#### Stimuli

The music used for the two public demonstration events was identical. As the target audience was taken from a broad section of the general public, the tempo-annotated music library contained 150 pop songs selected from recent commercial radio charts. Only songs with a basic 4/4 rhythmical pattern and a clear main audible beat were included. For any given gait frequency between 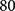 SPM and 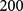 SPM, several songs were available. The volume of participants' headphones was limited to 75 dBA.

#### Task

The task given to participants was to freely experiment with the demonstration system. The experiment/demonstration was unrestricted in terms of walking speed and time. Participants were able to select a new treadmill speed or stop at will. The underlying experimental goals of the researchers were not disclosed to the participants prior to the experiment, and the participants were told that the prototype shown was for demonstration purposes. After the experiment, the participants completed a very short anonymous questionnaire requesting general information such as age, gender and it invited them to note down their experience with the system. After filling in the questionnaire, the participants also provided written consent allowing us to use the resulting data for research purposes and further development of the system.

#### Results

**Figure 5:**
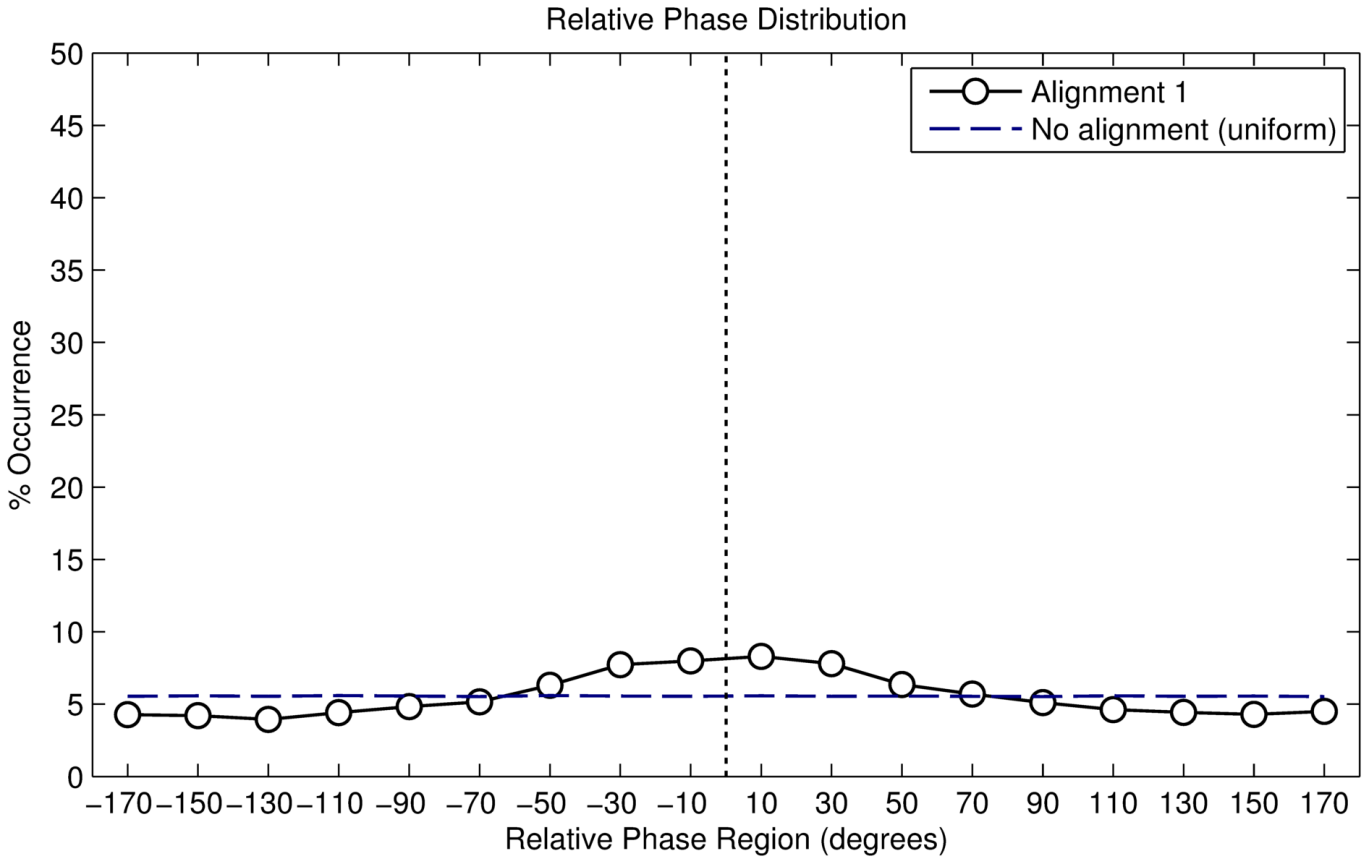
Angular histogram of alignment strategy 1 (period-adaptive phase-random).

In total, we recorded 51689 valid right footfalls or steps. The resulting distribution is described in Table 2, the angular histogram is shown in Fig. 5. The histogram shows a clustering of phase angles around 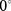, while a minor cluster is visible around 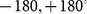. The distribution can therefore be interpreted as bimodal. We found that the distribution is not uniformly dispersed (Omnibus test for non-uniformity, 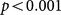). However, because the secondary cluster - around 180° - is barely visible, we can also interpret the distribution as unimodal. In this case, we find a statistically significant mean phase angle of 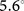 (Rayleigh 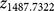, 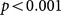).

**Table 2:** Distribution description.

Alignment Strategy	1	2	3 (0°)	3 (180°)	4
Mean angle 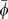	5.6°	4.7°	4.7°	−175°	−0.14°
High Limit (95% CI) 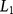	7.6°	6.5°	5.4°	−173°	0.004°
Low Limit (95% CI) 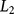	3.5°	3.0°	4.1°	−176°	−0.29°
Resultant vector length 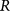	0.17	0.27	0.73	0.44	0.92
Circular Variance 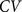	0.83	0.73	0.27	0.56	0.08
Angular deviation 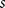	1.30	1.2	0.73	1.1	0.4
Circular skewness 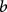	0.015	0.0095	0.042	−0.08	0.024
Circular kurtosis 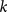	0.066	0.13	0.52	0.24	0.84

Both results confirm our hypothesis, namely, that the music-to-movement alignment algorithm has a significant influence on the participants' behaviour. Had this not been the case, we would have seen a uniform distribution. The resulting vector length 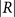, or measure of synchrony, equals 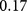. This indicates a higher concentration of angles around the circular mean.

**Figure 6:**
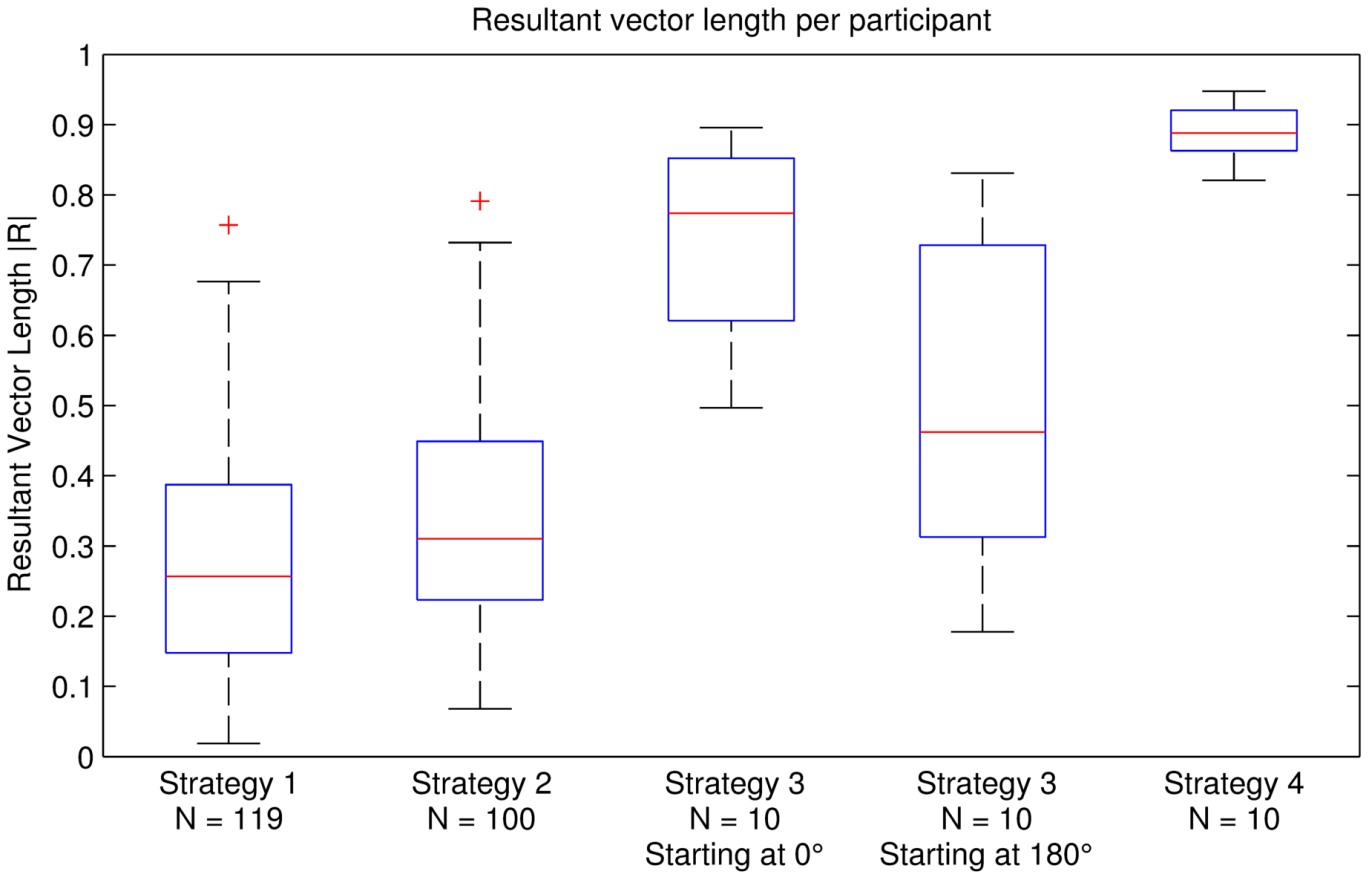
Resultant vector lengths of individual participants for all alignment strategies.

Finally, individual analysis showed that 71.8% of the 119 participants had a significant mean angle, determined using the Rayleigh test. The individual resultant vector lengths are shown in Fig. 6.

#### Discussion

**Figure 7:**
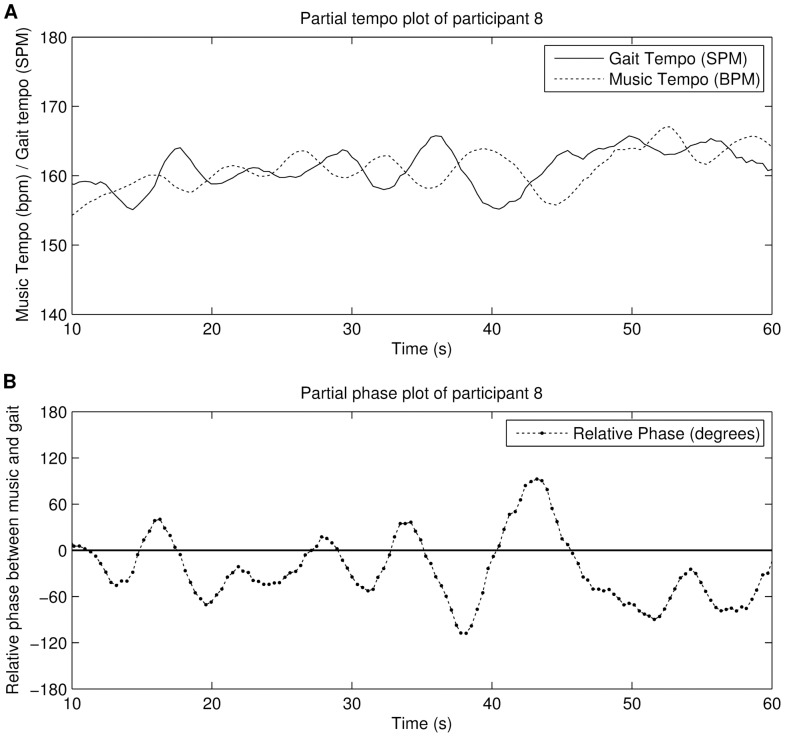
Excerpt from the experiment of alignment strategy 1 (period-adaptive phase-random). Figure A shows the tempo plot, figure B shows the phase evolution per step. Figure A and B show the wobbling pattern often found in the data.

The results show that the distribution is not uniform, with a slightly higher concentration of phase angles around 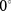. However, while the increase is noteworthy, we expected a higher concentration around 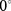 (i.e. more steps close to the beats). Several factors may be account for this. We should first consider the instability of the human gait: it rarely remains constant and the average frequency continuously fluctuates around a mean. Therefore, 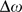 never really converges to zero, in contrast with what was expected, due to the averaging of the gait frequency. A second factor is that the interaction is unstable in the sense that a music tempo and the gait tempo tend to “wobble” or alternate around the mean. See Fig. 7A and 7B for an illustration of this phenomenon. This wobbling may be a consequence of the used gait tempo; a byproduct resulting from the following method. Music adjustment lags several seconds behind, the result being a loss of the phase alignment with every small period change. An in-depth data analysis reveals that participants subsequently try to readjust their pace in an attempt to get back in time with the music. However, the system reacts again to this change and phase alignment is again lost. This pattern often repeats itself and therefore the interaction seldom stabilises, as illustrated in Fig. 7A and 7B. A larger step detection window might solve this problem but that would result in more stable music stimuli, which is the topic of our second alignment strategy. A third plausible factor for the lower synchronisation rate is the restriction imposed by the treadmill. This device may restrict the movement of the participant [51]. However, he or she is still free to change gait parameters for any given speed, most notably the step length and thus the steps per minute. However, it can be argued that this action might feel somewhat unnatural.

#### Conclusion

To sum up, the results show that alignment strategy 1 has indeed an influence on the gait of the user. More steps were synchronised than unsynchronised, but the strategy suffers either from very low phase stability or high circular variance. The results suggested making a change in the alignment strategies such that the interaction would increase stability.

### Alignment strategy 2: period-fixed phase-random

#### Algorithm description

In this alignment strategy, the music period is kept stable once it has been chosen. This is done by playing the music back using a tempo that corresponds to observed gait tempo. Similar to the first alignment strategy, the relative phase is not used in the alignment, as shown in Fig. 4. After a song is finished, a new song is selected based on the most recent measurement of the gait tempo. The pseudo-code found in table 3 describes the alignment strategy and its parameters.

**Table 3:** Algorithm 2.

Pseudocode alignment strategy 2
1: **for all** steps **do**
2: 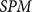 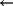 average gait frequency of last 5 steps
3: **if** current song ending **then**
4: choose new song ( 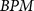 as close to 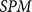 as possible)
5: MusicTempoModifier ( 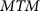 ) 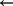 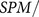 new 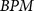 (change tempo only on song change)
6: **end if**
7: **end for**

#### Hypothesis

We assumed that alignment strategy 2 would be more stable than alignment strategy 1 because the musical tempo is kept stable once the playback starts. This would make it easier to synchronise to, thereby establishing a stable synchronisation interaction. We assume that if the strategy has an influence on the participants behaviour, then the resulting distribution of the relative phase will be different from a uniform (random) distribution. In addition, we foresee a statistically significant mean phase angle close to 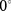, indicating that more steps were taken close to the main beat.

#### Participants

The experiment took place in the Czech Republic at the Univerzita Hradec Králové, where 112 students voluntarily participated in the experiment. However, 12 students did not complete the task successfully or had bad data and so were discounted in the further analysis. This resulted in the inclusion of 100 valid participants (age  = 20.2 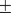 0.8 STD, 56 male, 44 female) in the analysis.

#### Ethics statement

This study only involves behavioural knowledge gathered from people that walk in the street. The participants signed a consent declaration which stated that have participated in the experiment of their own free will, were informed in advance about the task, the procedure and the technology used for measurement. They had the opportunity to ask questions and agreed that recordings of their actions would be made. They agreed that the recorded data would be used for scientific and educational purposes, only. In accordance with the general standards at the Univerzita of Králové, and the faculty of Department of Management, their privacy is respected. Owing to the fact that the experiment was conducted in public spaces around the city Hradec Králové, permission from the municipality to conduct this study was required and given.

#### Setup

A mobile implementation was made allowing us to experiment with the alignment strategy while walking in an open field. The experimental setup consisted of a mobile implementation of the music synchronisation framework using the aforementioned music alignment strategy. The hardware used was a Samsung Galaxy Y (2011) phone, running Android 2.3. The device was equipped with inertial sensors used to capture step detection. The device was placed on a belt worn around the hip; steps could be detected using peak detection methods on the accelerometer signal of the axis, positioned perpendicular to the earth (where the hip absorbs the shock of the footfall, and changes direction from downwards to upwards). Participants listened to the provided stimuli using a Sennheiser HD62TV headphone. The device also logged all available information for later analysis.

#### Stimuli

Participating students were asked beforehand to submit their favourite songs. From this list, a selection of 40 songs was made, each with a tempi that fell within the typical walking range of 80 to 140 BPM. Additional selection criteria were then applied to achieve stimuli that are more or less similar to the previous experiment. This is, stimuli in 4/4 metre with a clearly audible main beat. As no phase vocoder was available on the mobile system, several versions of each song were created beforehand with varying tempi, namely −3%,−2%,−1%,0%, +1%, +2% and +3%. This resulted in an annotated library of 280 songs normally divided in the tempo range of 80 to 140 BPM. As the average human walking tempi in a natural setting is around 110–120 SPM [52], more songs were available in this range. Once a song had been played, all other versions of the song were removed from the library to prevent users would hearing the same song twice. Each song's intro was manually removed (if there was one) and then cropped to a duration of two minutes, resulting in a broad enough range of songs for the user, and which immediately began with an audible beat to encourage spontaneous entrainment. The volume of participants' headphones was limited to 75 dBA.

#### Task

The task for the participants was to follow a predetermined outdoor path of 1.8 km taken at their own preferred speed, while listening to music. The path was clearly marked using orange indicators on the ground, in addition to a map which they could study beforehand. The participants were not informed about the goal of the experiment, nor were they told to synchronise or adapt to the music. Instead, they were told that the experiment was about the perception of the environment during free walks. Individual spontaneous gait frequencies were determined during the first two minutes of the walk, without auditory feedback. After the initial phase, music was provided with tempi matched as close as possible to the users gait frequency. Each song excerpt played for two minutes, after which a new song was chosen, matched closely to the most recent gait frequency. Upon completion of the track, participants filled in a questionnaire about their experience and the music.

#### Results

**Figure 8:**
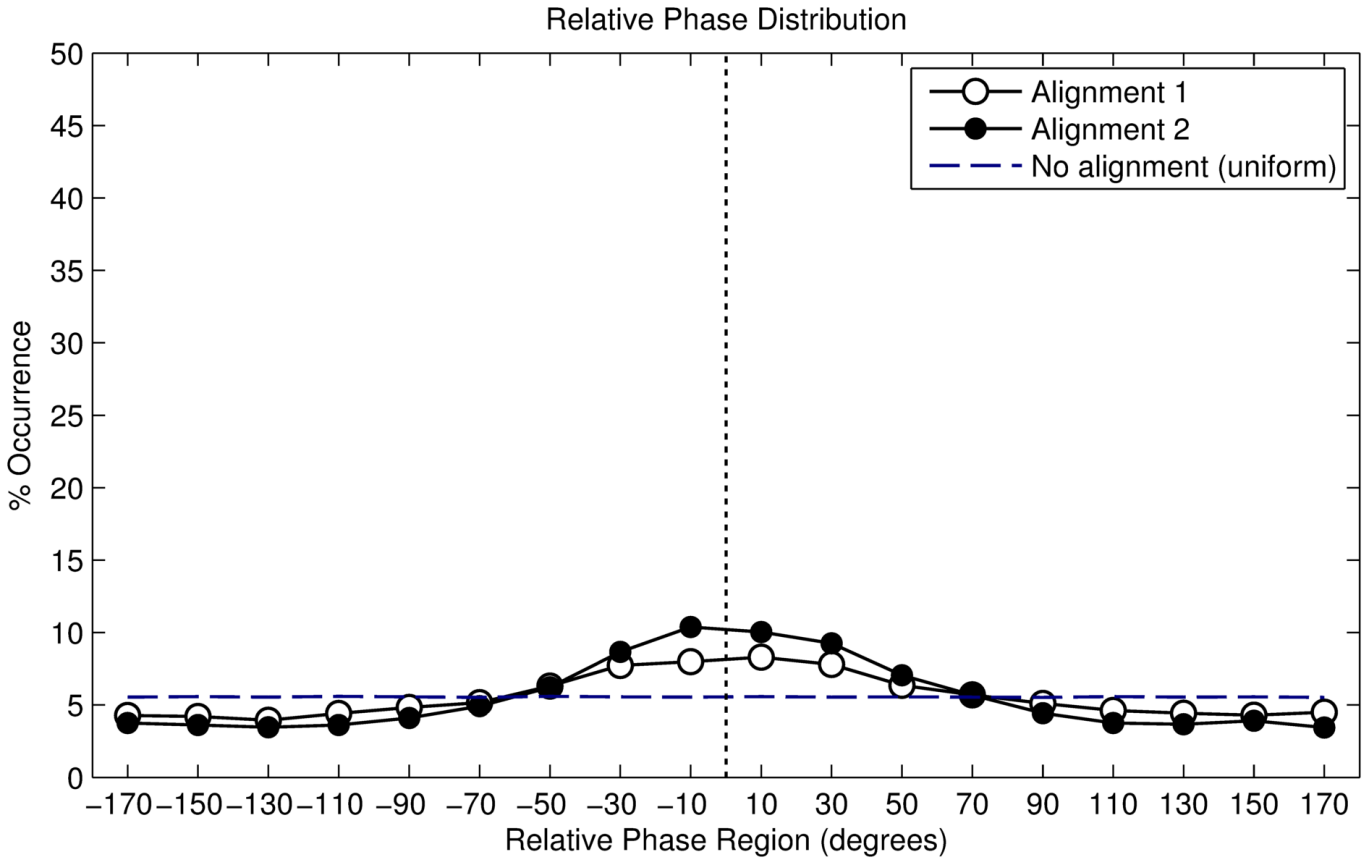
Angular histogram of alignment strategy 2 (period-fixed phase-random).

In total, we recorded 25,454 valid footfalls. The resulting distribution is described in Table 2, the angular histogram is shown in Fig. 8. The histogram shows a clustering of phase angles around 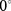. The distribution can therefore be interpreted as unimodal. We found that the distribution differs from a uniformly distributed one with a statistical significant mean phase angle of 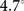 (Rayleigh 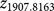, 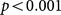).

The results illustrate that the alignment strategy has a significant influence on the participants' behaviour because otherwise we would have seen a uniform distribution. The resulting vector length 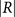, equals 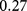, which indicates a higher concentration of angles around the circular mean and it also hints at a higher synchronisation rate than our previous alignment strategy.

Finally, individual analysis showed that 89.0% of the 100 participants had a significant mean angle, determined using the Rayleigh test. The individual resultant vector lengths are shown in Fig. 6.

#### Discussion

**Figure 9:**
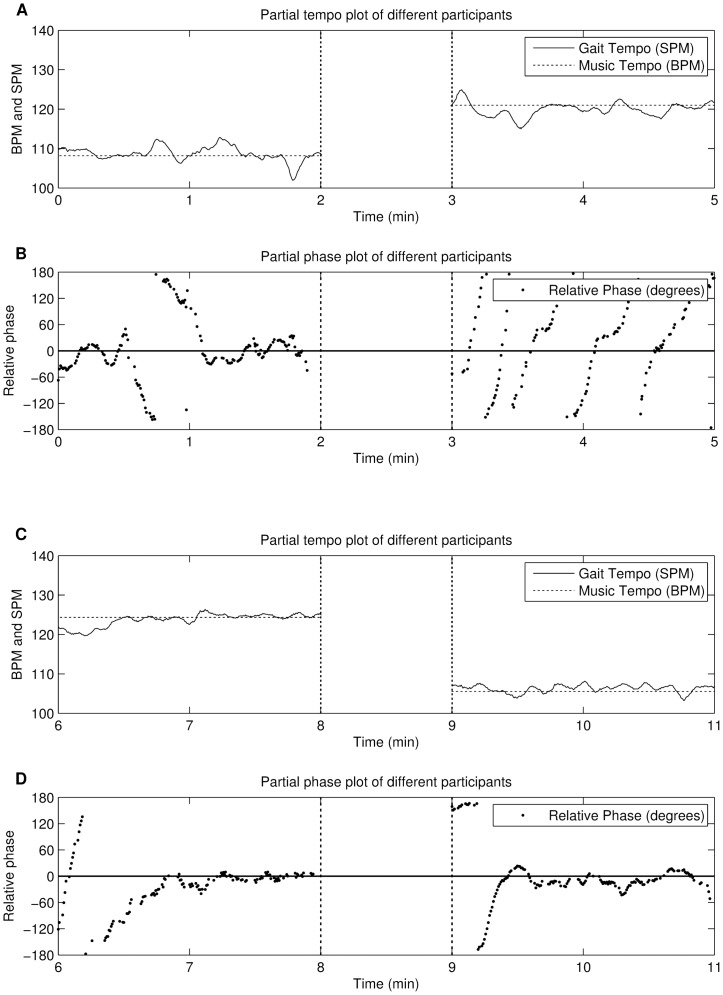
Excerpts from the experiment of alignment strategy 2 (period-fixed phase-random). Figure A and C show tempo plots, figures B and D show the phase evolution per step. The figure shows several examples of recurring patterns throughout the experiment.

The relative phase distribution shows the same pattern as our previous experiment, although a slight improvement in the distribution of the relative phase over the previous algorithm is noticeable. However, the in-depth data-analysis of individual relative phases and tempo plots reveals interesting recurring patterns. Fig. 9 shows four examples of different participants. The first example (Fig. 9, A and B, 0–2 min) shows initial phase synchronisation which is momentarily lost, but the participant eventually falls back in sync with the music again for the remainder of the song. The second example (Fig. 9, A and B, 3–5 min) shows that the participant does not appear to adjust to the stimuli. However, each time the steps coincide with the beat of the music, the participant keeps the same relative phase region for a few steps and gets temporarily “locked” in-phase. However, the participant is not permanently held, and can continue on in their own preferred locomotor tempi. By the end of the song however, the participants gait period is matched more closely to the music. The third example (Fig. 9, C and D, 6–8 min) shows that there is initially no synchronisation between participant and music due to a tempo difference. However, once close to the in-phase range, the user gets caught up by the main attractor and remains in phase for the remainder of the song. The fourth example (Fig. 9, C and D, 9–11 min) shows initial anti-phase synchronisation when the song starts, and this is maintained for several steps. However, after a while the participant gets locked into the beat and remains in-phase for the remainder of the song. The data suggests that the music has a certiain attraction force: once in-phase synchrony is obtained, the synchronisation was usually maintained until the end of the song. This observation became the starting point for our next alignment strategy.

We also observe that the initial music tempo selection is often several BPMs off target. This is due to the fact that gait frequencies tend to be very variable on their own. During the five seconds needed for music player adaptation, the actual gait frequency will most likely be changed and the music selection will no longer be optimal. This problem is inherent with real-time step detection, and especially when the music player is non-adaptive.

Another disadvantage of this alignment strategy is its insensitivity to sudden gait period changes. These occur frequently in our test due to environmental factors such as road crossings (although these were limited to a minimum), obstacles such as other pedestrians or participants looking and stopping to find the next directional marker. In such cases the new gait period rarely matched the gait period before the obstacle, creating a substantial difference between music and gait period, and thus, less synchronisation.

#### Conclusion

To sum up, the results show that alignment strategy 2 has an influence on the gait of the user. The algorithm facilitates entrainment and the resulting synchronisation is more stable than alignment strategy 1. We observed interesting recurring patterns in the data, most notably that once phase lock was obtained, it was seldom lost. We also noted two disadvantages to the alignment strategy. First, due to the gait frequency variability and the delay in the step detection, non-optimal stimuli was sometimes selected (based on old SPM values), thus lowering the chance of synchronisation and dragging down stability means. Secondly, the proposed method is impervious to large gait period changes. Improvements can be made by considering an in-phase lock at the start of a song, and by the re-introduction of period-adaptation.

### Alignment strategy 3: period-adaptive phase-starting 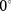


#### Description

This alignment strategy takes the relative phase into account, as well as the period, as shown in Fig. 4. Music is played with an adaptive period based on the average gait period of the last 5 seconds, which is identical to the first alignment strategy. In addition, this alignment strategy introduces a song start based on the users gait phase. The song starts playing when the relative phase is equal to the desired starting phase. Based on our findings in alignment three, we tested both a 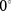 starting phase to maximise stability and a 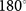 starting phase for comparison. The pseudocode found in table 4 describes the alignment strategy and its parameters.

**Table 4:** Algorithm 3.

Pseudocode alignment strategy 3 (tempo-adaptive phase-starting 0°)
1: **for** all steps **do**
2: 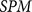 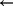 average gait frequency of 5 last steps
3: MusicTempoModifier ( 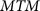 ) 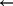 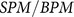
4: **if** 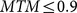 **or** 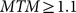 **then** (Tempo outside 10% stretch)
5: **if** for more as 5 seconds **then** (Avoid too many song changes)
6: queue new song ( 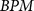 as close to 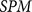 as possible)
7: **end if**
8: **end if**
9: **if** song in queue **then**
10: Skip to first beat in song
11: Start playing song when desired phase is reached (Calculate based on inter-steptime)
12: **end if**
13: **end for**

#### Hypothesis

For both the 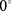 and 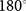 starting phase, we assumed that the alignment strategy would have an influence on the participants' behaviour. In particular, we expected that both resulting distributions would be different from a uniform (random) distribution, with a statistically significant mean phase angle, close to the starting phase of either 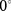 or 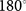, indicating that more steps were taken close to the main beat or off-beat. Furthermore, for the 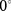 condition we expected a more stable interaction resulting in more footsteps taken in synchrony with the music compared to the previous alignment strategies. Finally, a different behaviour was expected when the starting phase was set to 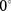 versus 180°. This included a higher enjoyability for the 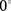 starting phase and a lower resultant vector length for the 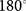 starting phase (i.e. more deviation from the starting phase because of the lower attraction force of the offbeat phase compared to the main beat). We also expected a difference in performance (the distance travelled).

#### Ethics statement

The subjects signed a consent declaration which states that they are freely participating in the experiment, have been informed in advance about the task, the procedure and the technology used. They were given the opportunity to ask questions and agreed that anonymous recordings of their actions would be made for scientific purposes, only. In accordance with the general standards set by our university and our faculty, security was guaranteed (the indoor task carries no risk), and privacy is respected. According to Belgian law for experiments aimed at research performed to further the development of biological or medical knowledge (cf. 7 May 2004 Law concerning experiments on the human person (Ch.II, Art.2, Par.11)), means our research is exempt from the requirement to obtain ethical approval because the study only involves behavioural knowledge.

#### Participants

The experiment took place in Belgium at the University of Ghent. In total, 12 participants (mean age  = 21.2 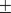
1.7, all female) volunteered for the experiment. Two participants did not complete the trial; all the recorded data for the remaining 10 participants could be used in the analysis. After a short explanation of the treadmill's operation and safety mechanism was given, participants could freely walk or run on the treadmill. Once the participant was comfortable with the treadmill and its operation, the headphones were given, and the data capture and auditory feedback was set in motion.

#### Setup

The experimental setup was similar to the first experiment: a treadmill, a computer running the music framework with the above algorithm and a Sennheiser HD62TV headphone. Step detection was done using a pressure sensor located below the front of the treadmill. The resulting signal indicated the weight in front of the treadmill, where a sharp increase indicated a footfall. This resulted in very accurate gait phase information, a necessary item for this experimental setup.

#### Task

Participants were asked to freely walk and run on a treadmill for 15 minutes. Each participant tested the system twice, but not on the same day, in counterbalanced order. The only difference between both trials was the starting phase: either 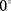 or 180°. After each trial, a short survey about the physical enjoyment during the exercise was filled in by the participants. For this, we used the Physical Activity Enjoyment Scale (PACES) [53], a questionnaire of 17 questions to be answered on a 7-point Likert scale. The PACES scale is verified with a similar population [54]. Participants were free to change the treadmill speed as they wished. Again, they were not informed about the underlying synchronisation goal nor the differences between both conditions, but were instead told the experiment was about the subjective enjoyment experienced when walking or running on music.

#### Stimuli

The music used for both trials was identical to the first experiment where we tested the tempo-adaptive strategy. That is, 150 pop songs selected from recent commercial radio charts, with a 4/4 rhythmical pattern and a clear main audible beat. For any given gait frequency between 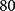 SPM and 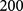 SPM, several songs were available. The volume of participants headphones was limited to 75 dBA.

#### Results for the 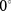 starting phase

**Figure 10:**
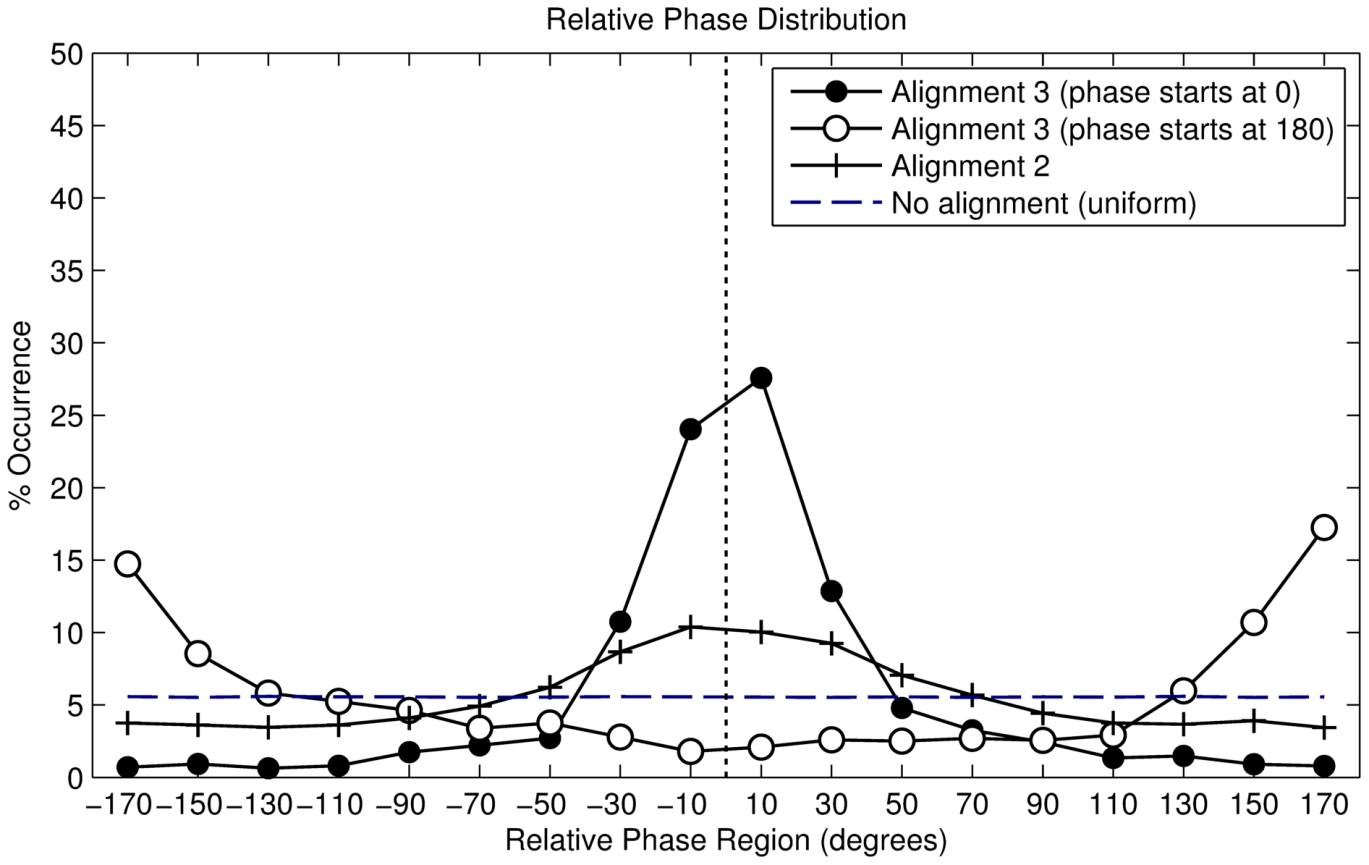
Angular histogram of alignment strategy 3 (period-adaptive phase-starting).

In total, we recorded 19,875 valid footfalls. The resulting distribution is described in table 2, the angular histogram is shown in Fig. 10. The histogram shows a clustering of phase angles of around 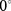. The distribution can therefore be interpreted as unimodal. We found that the distribution is not uniformly distributed with a statistical significant mean phase angle of 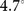 (Rayleigh 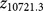, 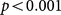).

The alignment strategy has a significant influence on the participants' behaviour, if this had not been the case we would have seen a uniform distribution. The resulting vector length 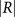, equals 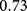. This indicates a high concentration of angles around the circular mean and also hints at a higher synchronisation rate than in our previous alignment strategies. Finally, individual analysis showed that all of the 10 participants had a significant mean angle, determined using the Rayleigh test. The individual resultant vector lengths are shown in Fig. 6.

#### Results for the 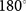 starting phase

In total, we recorded 20,348 valid footfalls. The resulting distribution is described in Table 2, the angular histogram is shown in Fig. 10. The histogram shows a clustering of phase angles of around 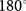. The distribution can therefore be interpreted as unimodal. We found that the distribution is not uniformly distributed with a statistical significant mean phase angle of 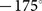 (Rayleigh 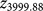, 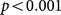).

The alignment strategy has a significant influence on the participants' behaviour, if this had not been the case we would have seen a uniform distribution. The resulting vector length 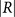, equals 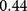. Finally, individual analysis showed that all of the 10 participants had a significant mean angle, determined using the Rayleigh test. The individual resultant vector lengths are shown in Fig. 6.

#### Comparing the 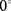 and 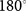 starting phase conditions

We compared both distributions using the Watson's U2 test, which computes the probability that, according to the null hypothesis, 2 samples of circular data come from the same population, or from 2 populations that have the same direction [55]. Analysis reveals the distributions differ significantly (Watsons 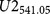, 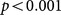), indicating that a different starting phase has an effect on the participants walking behaviour. We note that the resultant vector length is much higher for the 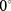 condition (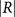  = 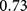) than the 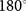 condition (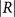  = 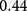), indicating that participants deviated more from the 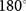 starting phase. There was a significant difference in the PACES scores for 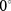 starting phase (Mean  = 95.6; SD  = 10.6) and the 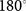 starting phase (Mean  = 87.9; SD  = 13.2) conditions; t(9)  = 2.37, p = 0.042. There was no significant difference in the distance travelled for 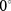 starting phase (Mean  = 1650 m; SD  = 314) and the 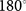 starting phase (Mean  = 1630 m; SD  = 261) conditions; t(9)  = 0.347, p
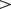
0.5.

#### Discussion

**Figure 11:**
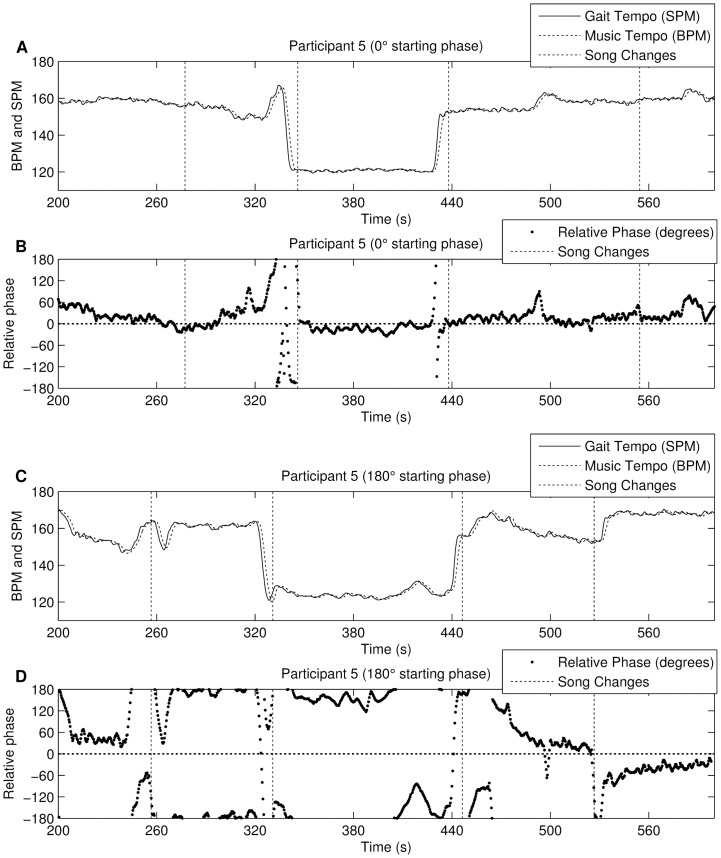
Excerpts from the same participant using alignment strategy 3 (period-adaptive phase-starting). A and B show the 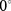 starting phase, C and D show the 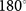 starting phase condition from the experiment using the phase-aware stimuli. Figures A and C show tempo plots, figures B and D show the phase evolution per step. The examples show the influence of the starting phase: when starting in sync, the phase lock is mostly maintained while in the offbeat condition, there is a higher chance of deviating from the starting phase towards the 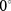 phase.

The likelihood of entraining with the music, using the third strategy, is much higher compared to random music stimuli (i.e. a random playlist with various BPM values), and even the other alignment strategies. Yet, the only difference with alignment strategy 1 is a fixed starting relative phase angle. The best synchronisation is obtained when starting in the 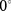 phase angle at the start of the song. As mentioned in the discussion of strategy 2, participants do seem to remain in phase once a low relative phase angle is obtained (see Fig. 11 A and B). This alignment, specifically the 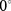 start angle, therefore appears to be very effective when trying to encourage stable synchronisation. Participants react differently with the 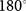 condition. Fig. 11 C and D shows a 7 minute excerpt from the experiment. In this case, the participant seems to have two reactions: either try to go in-phase (e.g. 440 s–530 s), which seems a conscious effort by slowing down/speeding up, or remaining in anti-phase (e.g. 330 s–440 s). When starting in 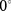 however, one rarely goes to the 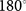 starting phase. This indicates that the 0 degree attractor is stronger than the 180 degree. Fig. 6 shows the resultant vector length distribution (calculated per person). The 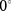 condition has a higher mean resultant vector and lower standard deviation than the 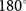 condition. This indicates that the 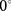 starting phase results in a more stable interaction with the system. Lastly, the PACES score (physical enjoyment) was significantly higher in 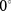 starting phase, indicating a higher enjoyment when running in phase-synchrony. Participants kept a more stable pace and had less tempo changes, i.e. participants were more likely to maintain the same pace when the musical beat corresponded with the footfall - and started in-phase - than when the music started in anti-phase. When starting in anti-phase, some participants sought to get in sync, but while doing so, they changed gait frequency and thus the system resets itself to 180 degrees. This ‘frustration’ could have caused the lower PACES. However, it did not influence the total distance travelled (the performance).

Note that this study has a lower participant number than previous studies (N = 10). Furthermore, the participants were all female, which makes it difficult to generalise about the results in relation to a larger population. However, the effects are so significant that we believe that this alignment algorithm will have similar results on a larger population.

#### Conclusion

To sum up, the overall data and examples show that the likelihood of having a stable synchronisation is very high when using the 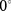 starting phase compared to the previous strategies. When a new song was chosen, participants started in phase, which resulted in a much more stable synchronisation. The proposed alignment strategy does seem to solve the problem of finding the in-phase alignment that affected the previous alignment strategies. However, there will always be people who are unable to synchronise. For them there is the option to go one step further by using both phase-adaptation and a period-adaptation.

### Alignment strategy 4: period-adaptive and phase-adaptive to 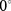


#### Algorithm description

This alignment strategy adapts the relative phase analysis continuously, as shown in Fig. 4. This happens when the allowed synchronisation range from −30° to 30° is exceeded. If this deviation persists, then an extra period adjustment is applied to enable the phase alignment to converge towards the 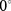 relative phase angle. The pseudocode in table 5 describes the alignment strategy and its parameters in more precise detail.

**Table 5:** Algorithm 4.

Pseudocode alignment strategy 4 (tempo-adaptive phase-adaptive to 0°)
1: **for** all steps **do**
2: 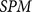 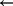 average gait frequency of 5 last steps
3: 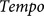 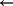 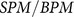
4: **if** Tempo 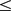 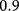 **or** Tempo 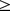 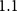 **then** (Tempo outside 10% stretch)
5: **if** for more as 5 seconds **then** (Avoid too many song changes)
6: Queue new song ( 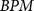 as close to 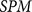 as possible)
7: **end if**
8: **end if**
9: **if** Phase 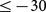 **then** (Step after beat: speed music up a bit)
10: Tempo 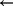 Tempo + 0.03
11: **else if** Phase 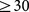 **then** (Step before beat: slow music down a bit)
12: Tempo 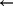 Tempo - 0.03
13: **end if**
14: MusicTempoModifier 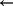 Tempo (Set the final music tempo)
15: **if** song in queue **then**
16: Skip to first beat in song
17: Start song when desired phase is reached (Calculate based on inter-steptime)
18: **end if**
19: **end for**

#### Hypothesis

In this alignment strategy the phase and the period are continuously adapted to the participants' phase and period. Accordingly, by definition, the participant will be synchronised with the music. If the technology works, there should be a statistically significant mean phase angle, close to 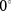. As a matter of fact, there will be more footsteps in synchrony with the music compared to all previous alignment strategies.

#### Ethics statement

The subjects were fully informed of any risk associated with the experiments and each underwent a medical check-up ahead of any testing, and before giving their written consent for participation. The study was approved by the ethics committee at the University of Ghent Hospital. Procedures were followed in accordance with the Helsinki Declaration recommendations.

#### Participants

The experiment took place in Belgium at the Department of Movement and Sports Sciences of Ghent University. 12 students from Physical Education and Movement Sciences (Ghent University) were recruited for the study participation on a voluntary basis. All participants were recreational but non-professional runners. The experiment took place in the context of another experiment where we tried to determine whether there is a difference in ergogenic parameters (such as blood pressure, heart rate, lactate, oxygen consumption, etc.) when training on phase-synchronised music versus regular (unsynchronised and random) music. However, the physiological results are not reported in the present paper. Proper safety precautions were taken due to the high intensity of the tests. A large red panic button that automatically stops the treadmill was made easily accessible, and participants wore a safety harness to prevent them from hitting the ground in case of a fall. A doctor was present during the VO2 max tests, to examine the participants beforehand and judge them fit for the tests. After the completion of all the trials, participants were rewarded with a 5 euro shopping card gift voucher. 2 of the 12 participants dropped out due to injuries unrelated to the experiment. 10 participants (age 23 +- 3 SD; 6 female, 4 male) were included in the analysis. Only the two (anaerobic and aerobic) synchronous music conditions are used for further analysis, as only those are relevant in this context.

#### Setup

The experimental setup consisted of a professional treadmill, a computer running the music framework with the above algorithm and a Sennheiser HD62TV headphone. Step detection was done using two iPods, located at the participants' ankles, streaming accelerometer and gyroscope data wirelessly to the computer at 100 Hz. Steps were detectable by analysing both the gyroscope and accelerometer signal. By using the gyroscope, forward to backward leg movement was detected, which occurs a fraction prior to the footfall. The gyroscope signal was used to predict a small footfall window 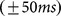 in which the first peak in the accelerometer signal from the axis perpendicular to the earth was denoted the footfall. This method has proven to be very accurate as no more dubious peaks were detected.

#### Task

The task consisted of 6 thirty-minute sessions of running on a treadmill, and a pretest. The pretest comprised a BRMI2-test used to determine individual motivational music. This was followed by a VO2-Max test in order to determine individual treadmill speeds for the anaerobic and aerobic threshold. A medical doctor was also present during these tests. Two treadmill speeds were thus determined: a high-intensity speed and a medium-intensity speed. For each speed, three musical conditions were tested: no music, random music with a tempi of around 120 BPM (thus no synchronisation would be possible) and the forced phase synchronisation music. The six conditions were tested in counterbalanced order. The trials were spread evenly over three weeks, a break of at least 48 hours was given between each session to allow the participant to recover. Participants were also asked to follow the same lifestyle pattern and perform the test at roughly the same time of day for each test. Participants were instructed to run for thirty minutes at a given treadmill speed. They were informed that the study concerned the influence of music on running, but were not informed, beforehand, of the different conditions. The secondary goal for evaluating the relative phase distribution was also not mentioned.

#### Stimuli

A preselection of 100 songs from the pop music repertoire was made by the experiment supervisors. Before the VO2-Max pretest, each participant rated all 100 songs using the BMRI2 questionnaire/survey [56]. The BMRI2 is a scale used to assess the motivational power a song has upon a participant. This resulted in two playlists per individual, one for the synchronised condition (150–200 BPM) and one for the regular condition (110–130 BPM), containing only motivational songs. The volume of participants' headphones was limited to 75 dBA.

#### Results

**Figure 12:**
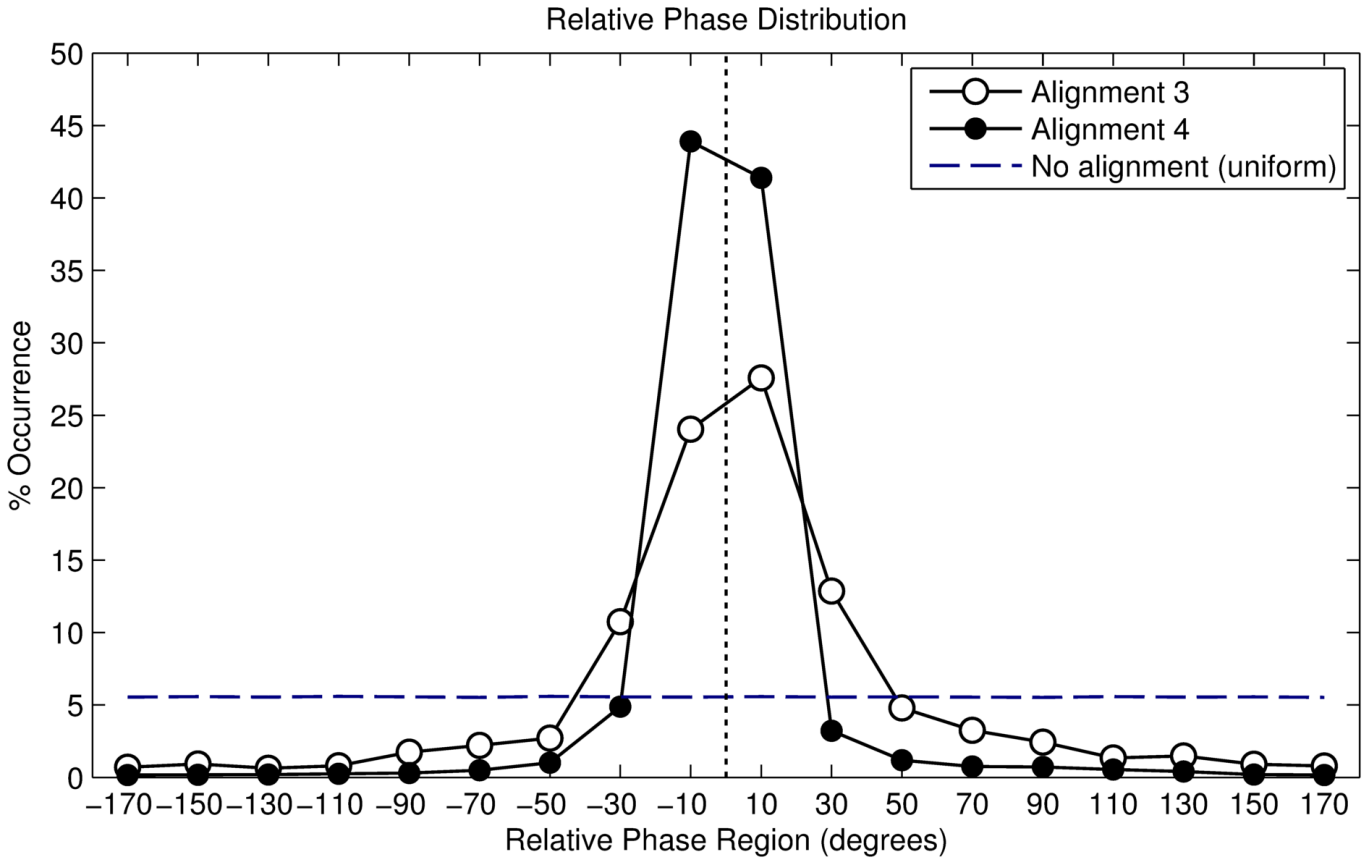
Angular histogram of alignment strategy 4 (tempo-adaptive phase-adaptive).

In total, we recorded 104334 valid footfalls. The resulting distribution is shown in Table 2, the angular histogram is shown in Fig. 12. The histogram shows a clustering of phase angles around 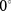. The distribution can therefore be interpreted as unimodal. We found that the distribution is not uniformly distributed with a statistical significant mean phase angle of 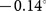 (Rayleigh 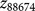, 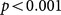). The results confirm the hypothesis that this alignment algorithm shows the best results in terms of synchronisation. The resulting vector length 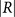, equals 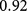. This indicates a high concentration of angles around the circular mean and also hints that this is the highest synchronisation rate out of all of the alignment strategies. Finally, individual analysis showed that all of the 10 participants had a significant mean angle, determined using the Rayleigh test. The individual resultant vector lengths are shown in Fig. 6.

#### Discussion

**Figure 13:**
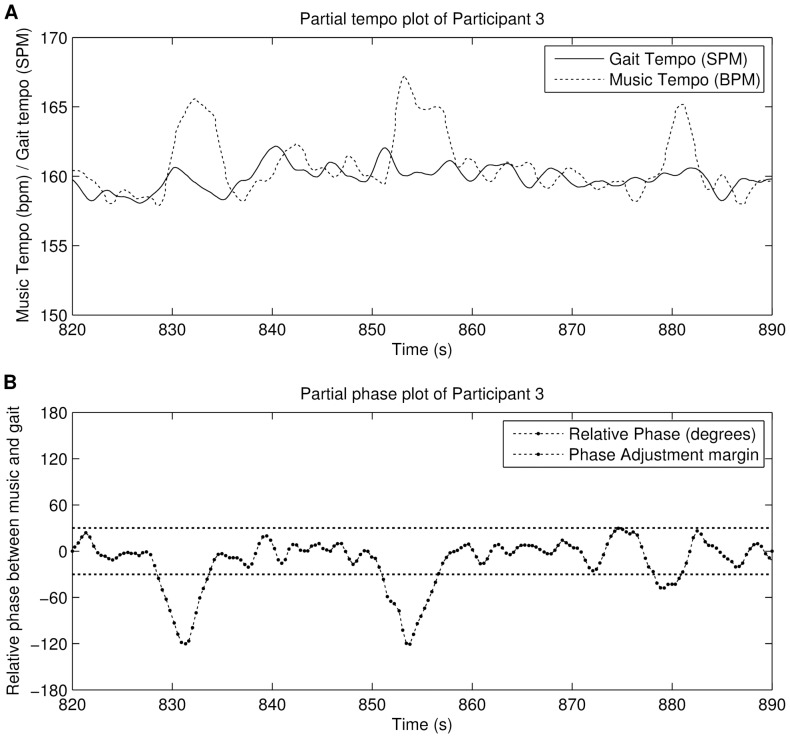
Excerpt from one participant during the experiment using alignment strategy 4 (tempo-adaptive phase-adaptive). Figure A shows the tempo plots, where the tempo adaptation from the algorithm is clearly visible. Figure B shows the relative phase.

The results show that the alignment strategy is very effective in maintaining synchronisation. Steps taken outside the synchronous range are automatically corrected, although due to the delay caused by the step detection, it may take a few seconds to give an effect. Although some of the steps included abrupt step length changes (due to experimentation of the participants) and small pauses made to take blood samples from the participant, this had little or no effect on the outcome. It is also logical that almost no steps were taken in off-beat synchronisation, because the alignment algorithm tries to push participants towards the in-phase angles. Fig. 13 shows the effect of the alignment strategy on the phase angle. The bottom graph includes three lines: 0°, −30°and +30°. The latter two are the boundaries from which the system starts adjusting the music tempo in order to manipulate the relative phase. It is clear that, once the phase angle steps outside of the boundary, the music tempo chances 3% to push the relative phase back to 0°. We sometimes observe a small after effect, in the sense that the adjustment results in a reverse tempo adaptation (slowing the music down after accelerating) in order to minimise the relative phase. This is a recurring pattern, which indicates that by tweaking the arbitrary chosen parameters, the algorithm could be improved. In that regard, both systems influence each other; and would thus qualify as a form of entrainment. Interestingly, with this alignment strategy there is basically no human entrainment to get in-phase. Instead, the entrainment is done by the music player. What is left for the participant is “being in-phase”, without “getting in-phase”. This effect requires further study as some participants reported that this type of synchronisation felt rather synthetic and mechanical. We do believe that a fine-tuning of this alignment strategy may lead to even more stability and decrease the mechanical feeling of the system. For example, the values −30°and 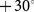 were somewhat artificially chosen and they could be optimised. In addition, a sudden 3% shift in tempo might be noticeable to users.

#### Conclusion

As expected, alignment strategy 4 has the highest amount of steps that were in synchrony with the musical beat. The main reason is that the music player adapts to human movement so that there is a constant in-phase situation. However, more study is needed to consider the psychological effects of this type of alignment. These issues will be addressed in further research, where we will also address the optimisation of this alignment strategy.

## General Results

Table 6 summarises the alignment strategies and the related experimental context for each strategy according to the following contextual parameters: period adaptation, phase adaptation, number of participants, age, setup, walking area, stimuli, tasks, duration, and total of recorded valid steps. Table 2 summarises the findings for spontaneous synchronisation for each experiment, according to the following statistical parameters: mean angle, upper 95% confidence limit, lower 95% confidence limit, resultant vector length 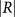, variance 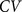, angular deviation 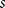, circular skewness 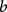 and circular kurtosis 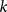. Table 7 gives a summary of the major results of this step-by step development of the alignment strategies. Finally, Fig. 6 shows the resultant vector lengths for individual participants.

**Table 6:** Overview of the four alignment strategies and related experiments.

Alignment Strategy	1	2	3	4
Period adaption	Adaptive	Fixed	Adaptive	Adaptive
Phase adaption	Random	Random	Fixed starting phase	Adaptive to 0°
Valid participants (N)	119	100	10	10
Participants Age	22 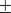 12	20 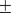 0.8	21 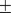 2	23 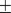 3
Setup	PC	Mobile	PC	PC
Walking area	Treadmill	Outdoor	Treadmill	Treadmill
Stimuli	pop selection	pop selection	pop selection	motivational pop
Tasks	Any tempo	Walk	Any Tempo	Run
Duration	Voluntary	2 km walk	15 minutes	30 minutes
Recorded valid steps	52050	29419	19875 (0°)	104334
			20348 (180°)	

**Table 7:** Effect of strategy on entrainment and alignment.

Alignment strategy	Effect on entrainment and alignment
1	Person entrains to phase, but with unstable alignment
2	Persons entrains to phase, difficult but with possibility of stable alignment
3	Person entrains to phase with stable alignment
4	Machine entrains to phase, no human entrainment, forced alignment

Alignment strategy 1 leads to a ‘wobbling behaviour’ or an unstable synchronisation. The reason being is that the person adapts to the system, probably in attempt to try to align themselves to find a stable relative phase (see Discussion below), but the systems interprets this as a period change and adjusts it accordingly. We should also mention that the environment in which the D-Jogger system was tried out, namely a demonstration during an exhibition, may have stimulated a person's explorative behaviour. The context may have stimulated an explorative attitude to test how D-Jogger would respond to different tempi. This explorative attitude could have had a negative effect on the stability of the synchronisation.

To overcome the wobbling effect, alignment strategy 2 uses a fixed period once the song is chosen. Note, this only occurs in this strategy where the tempo is not changed during the song. However, the phase is not controlled, which means that getting into the in-phase synchronisation is a task left to the participant. This strategy is successful once the participant accesses the stable phase synchronisation. However, getting in-phase appears more of a challenge than keeping the in-phase alignment. It should be noted that during the outdoor walk, there may have been some obstacles (e.g. stepping up and down a footpath, other people walking) that disturbed the regularity of the gait.

In order to facilitate the entrainment, alignment strategy 3 sets the music in-phase to begin at the start of a song. During the remainder of the song, the tempo is adaptive, while the relative phase is neglected. Although this experiment was conducted under laboratory conditions on a treadmill, it suggests a major improvement over the phase-random approach of alignment strategy 1. The main finding is that being in-phase at the beginning of the song helps to maintain stable synchronisation immediately. And when the tempo changes, the phase will also change, but it will remain in synchrony with the participant.

Alignment strategy 4 is a special case because the music player is now entirely adapted to the person. This implies that there is no entrainment on the part of the person, only alignment. If the person changes phase or period, then the music player will respond immediately.

To sum up, we can conclude that spontaneous human entrainment seems to be most effective in alignment strategy 3 for reasons that can be attributed to the in-phase lock at the beginning of the song. Interestingly, human entrainment to find the beat can be eliminated and replaced by machine entrainment, as shown in alignment strategy 4.

## Discussion

The results of the present paper suggest that the spontaneous entrainment between a person and music (D-Jogger) can be influenced by manipulating the period and the phase of the music. This can be understood in terms of coupled human-music rhythms, and phase-error correction mechanisms.

The dynamics of the coupled human-music rhythms can be schematically understood in terms of the attractor dynamics of two coupled oscillators [32], [33]. Both the human and the music player can be conceived as oscillating rhythms; whose timing can be measured at specific events, such as the footfall on the ground and the musical beat. Stable synchronisation is defined in terms of a timing difference between these events (relative phase). Similar to the dynamics in these models (known as HKB models), the dynamics of the coupled human-music rhythm is naturally drawn to an alignment with zero relative phase (0° attractor). However, the dynamics of two coupled rhythms also depends on the coupling interface, which functions as a mediator for the interaction. In our setup, this coupling interface consists of a music player, whose timing can be manipulated on the basis of different alignment strategies.

The results show that all strategies resulted in a significantly higher amount of steps in phase synchrony with the main beat ( = phase-lock), when compared to a random playlist. However, the strongest synchronisation was obtained with strategies involving period and phase manipulation, so that songs start in perfect synchrony. The weakest synchronisation was obtained with strategies involving period manipulation, but not phase manipulation. We assume that strategies that immediately lock the subject in-phase with the music (at the start of each song) are superior because they allow the subject to adopt a stable sensorimotor scheme (with phase-error correction mechanism) that predicts the beat more accurately right from the start of a song. Strategies that require the subject to find the in-phase lock are more difficult due to fluctuations in the interaction. Finding stable synchronisation requires more pull and push to effectively lock into a fluctuating attractor. If one injects too much effort, it may overshoot the balance, while injecting too little may result in a failure to attain the attractor. Instead, to keep stable synchronisation requires that once it is locked, some degree of tweaking and re-adjustment will be required to ensure it remains locked into the beat. In other words, less effort is needed to *keep* the alignment, than to *find* the alignment. Note that alignment strategy 4 not only eliminates the human task of finding the beat, it also eliminates the task of keeping the beat, and the subject stays aligned in-phase with the music.

The interacting rhythms should be seen as the result of a layered interplay of several factors. Our manipulations here are confined to timing only, leaving all other parameters of the music unaffected. However, due to the complex character of the human response, other factors may influence the entire interaction dynamics. Factors that determine the human rhythm are, among others, variability [57], motor resonance [7], and attending [27], [58]. Factors that determine the music rhythm are the intrinsic musical features that define the differences between songs, and the extrinsic manipulatory features that define how songs are played back using a music player. In light of the recent results produced by Leman et al. [4] by Fritz et al. [3], we are aware of the fact that musical style and physical exertion may have an strong motivational effect on music-movement interactions. Music style can either speed up or slow down walking velocity, even when the music has the same tempo. Although aspects of style and effort, as well as motivation in general, have not been considered in the present study, we believe that an interactive personal-music system opens up promising new possibilities in our understanding of how music empowers people.

### The influence of the starting phase

The coupled oscillator model suggests differences between the attraction force of the in-phase attractor and the anti-phase attractor [32], [33]. We have collected data on this difference from the third experiment, where we tested the period-adaptive phase-aware strategy. Recall that this alignment has a starting phase parameter, set to either 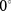 or 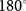. More steps were located in the starting attractor basin when the starting relative phase was 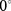, rather than when the starting relative phase was 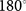. This shows that an attractor at 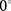 is much stronger than an attractor at 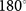. Moreover, participants were more likely to maintain the same pace when the musical beat corresponded with the footfall - and started in-phase - than when the music started in anti-phase. The difference between the 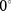 and 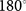 condition is also visible in the individual analysis (see Fig. 6). We also found that all participants had a significant mean angle (using the Rayleigh test), similar to the 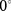 condition. However, due to the low number of participants in this experiment (N = 10, all female) more research is needed before generalisations can be made about these results.

### Limitations of our study

The development of D-Jogger is based on an approach where experiments provided feedback for further development and optimalisation. For example, the strategy taken with the adaptive phase was, at first, not present in the music player. It was developed and tested only after having realised that the period-adaptive strategy was insufficient, and was not as efficient as the fixed period. Nevertheless, we believe that the development of D-Jogger followed a logical path. As with the adaptive phase, after having first tested the period adaptation strategy, we then introduced the phase adaptation strategy. The results led to consistent general findings about entrainment and alignment. However, a major limitation to this approach is that each of the experiments cannot really be compared alongside each other. The experiments were placed in different contexts for different versions of the strategy. Moreover, there were exhibition conditions and laboratory conditions, and differences in the size of the groups tested. A follow-up study with a balanced control of conditions is needed to provide further comparison.

As to making comparisons of our work with the work of others, it should be noted that our emphasis on spontaneous entrainment is not the same as most of the walking-running studies that have been conducted thus far. When participants are requested to synchronise with music played back, by means of a non-adaptive music player, one typically finds that synchronous music has numerous advantages related to ergogenic, physiological and/or psychophysicological aspects [1], [6], [8]–[11]. This requires that the participant engages with the music in an intended way, which might alter the participants natural gait pattern. However, we believe that in many applications, instructed entrainment is not always realistic. We also believe that it is important to understand the subliminal effects of entrainment and alignment, but here too, further research is needed.

### Strengths of D-Jogger and open questions

The above results suggest that D-Jogger can serve as an assistive technology to motivate people to exercise physically. One application domain that has already been mentioned in the introduction is recreation, such as providing beat-locking during walking or running activities. However, D-Jogger can also be used as an assistive technology in the clinical context (e.g. as smart walkman for Parkinson patients), or for physical rehabilitation. Apparently, not everybody can synchronise to music, even when instructed to do so [4], [7], [59]. This group of people can be helped with a system that adapts to them. This shows the potential for an alignment strategy in which the entrainment problem is facilitated by the adaptability of the music player (alignment strategy 4). The merits of the strong, one-sided coupling are that there will always be synchronisation alignment, even if the user lacks a sense of rhythm or ignores the stimuli completely. Note that this approach makes objective comparisons between synchronised and unsynchronised music possible. For example, in endurance training literature, synchronised music requires participants to consciously synchronise to music and thus adapt their walking or running habits. This might result in biased comparisons as the participant, in order to synchronise to the music, under the controlled conditions may have run at a tempo that s/he would not have otherwise have run at. With this alignment strategy, one could determine whether there is a pure ergogenic or psychophysicological effect of the phase-synchronous music during exercise. In short, D-Jogger generates a number of new research questions such as: Which strategy is actually the best, and in what respect? What is the exact difference between spontaneous synchronisation and intended synchronisation in relation to performance? What is the role, the task, the context, the motivation, and the musical stimulus? D-Jogger thereby offers a new platform that allows such questions to be addressed in a manner that is straightforward.

## Conclusions

The goal of the present paper was to develop and optimise the alignment strategies of D-Jogger, an adaptive music player that facilitates the elicitation of spontaneous entrainment. We showed that different alignment strategies can indeed have an effect on spontaneous entrainment behaviour. We found that entrainment may be less effective if the attractor (zero relative phase) fluctuates (which is often the case in an interactive setup). The solution is that the relative phase should be set close to zero right from the start of the interaction. This “being in-phase” at the start seems to dramatically enhance the possibility of obtaining a stable synchronisation (“keeping in-phase”), as it reduces the effort of “finding in-phase”. From a theoretical point of view, this means that entrainment is most effective when it maintains a given stable alignment between movement and music. Finding this stable alignment is much harder to achieve.

The reason for wanting to produce an adaptive music player is that it would empower people to walk or run at their own pace, using a music player that automatically adapts to that tempo. In addition, there are several applications in the domain of assistive technologies and physical rehabilitation, even for people that cannot entrain to an external auditory stimulus. We believe that our technology and the implied results offer interesting perspectives for further study, providing useful applications in the area of sports and physical rehabilitation.
